# CSF N-Glycan Profiles to Investigate Biomarkers in Brain Developmental Disorders: Application to Leukodystrophies Related to eIF2B Mutations

**DOI:** 10.1371/journal.pone.0042688

**Published:** 2012-08-29

**Authors:** Anne Fogli, Christine Merle, Véronique Roussel, Raphael Schiffmann, Sylvie Ughetto, Manfred Theisen, Odile Boespflug-Tanguy

**Affiliations:** 1 Laboratoire GReD UMR INSERM U931 CNRS 6247, Faculté de Médecine, Clermont-Ferrand, France; 2 Service de Biochimie Médicale, CHU de Clermont-Ferrand, Clermont-Ferrand, France; 3 Proteodynamics, Biopôle Clermont-Limagne, Saint-Beauzire, France; 4 Institute of Metabolic Disease, Baylor Research Institute, Dallas, Texas, United States of America; 5 Département d'Information Médicale, CHU de Clermont-Ferrand, Clermont-Ferrand, France; 6 Assistance Publique-Hôpitaux de Paris, Hôpital Robert Debré, Service de Neuropédiatrie et Maladies Métaboliques, centre de référence « leucodystrophies », Paris, France; 7 Université Paris Diderot, Sorbonne Cité, Paris, France; University of Jaén, Spain

## Abstract

**Background:**

Primary or secondary abnormalities of glycosylation have been reported in various brain diseases. Decreased asialotransferrin to sialotransferrin ratio in cerebrospinal fluid (CSF) is a diagnostic marker of leukodystrophies related to mutations of genes encoding translation initiation factor, EIF2B. We investigated the CSF glycome of eIF2B-mutated patients and age-matched normal individuals in order to further characterize the glycosylation defect for possible use as a biomarker.

**Methodology/Principal Findings:**

We conducted a differential N-glycan analysis using MALDI-TOF/MS of permethylated N-glycans in CSF and plasma of controls and eIF2B-mutated patients. We found in control CSF that tri-antennary/bisecting and high mannose structures were highly represented in samples obtained between 1 to 5 years of age, whereas fucosylated, sialylated structures were predominant at later age. In CSF, but not in plasma, of eIF2B-mutated patient samples, we found increased relative intensity of bi-antennary structures and decreased tri-antennary/bisecting structures in N-glycan profiles. Four of these structures appeared to be biomarker candidates of glycomic profiles of eIF2B-related disorders.

**Conclusion:**

Our results suggest a dynamic development of normal CSF N-glycan profiles from high mannose type structures to complex sialylated structures that could be correlated with postnatal brain maturation. CSF N-glycome analysis shows relevant quantitative changes associated with eIF2B related disorders. This approach could be applied to other neurological disorders involving developmental gliogenesis/synaptogenesis abnormalities.

## Introduction

Genetic defects of N-glycan metabolisms due to abnormal hydrolysis (oligo saccharidosis), transport or storage (sialidosis and sialic acid storage disorders such as Salla disease and infantile sialic acid storage disease) and synthesis as in congenital disorders of glycosylation (CDG) are responsible for severe alterations of the CNS including myelination impairment [1]. Glycosylation is the most abundant post-translational event that yields functional active proteins. Glycan moieties play a major role for cell-cell and cell-matrix recognition during brain development and functions. Glycosylation maintains the axono-myelin-glial compartments in close contact with the blood and CSF compartments. Specific myelin glycoproteins such as MAG (myelin-associated glycoprotein), and MOG (myelin/oligodendrocyte glycoprotein) are respectively at the axonal and matrix interface [2]. Moreover, abnormal sugar chains of the cerebrospinal fluid (CSF) transferrin have been reported in various neurodegenerative disorders [3], [4]. Decreased CSF asialotransferrin to sialotransferrin ratio is considered as a biomarker of the CACH/VWM disorder (childhood ataxia with central nervous system hypomyelination/vanishing white matter) [5], [6] and can be measured using a HPLC method when 1.5 mL CSF is available [7]. This vacuolating form of leukodystrophy is related to mutations in the initiation factor, eIF2B, an ubiquitous factor involved in the global protein synthesis and its regulation under normal and stress conditions [8]–[12]. Its nucleotide guanine exchange activity (GEF) measured in patients transformed lymphocytes is decreased in eIF2B-mutated cells in comparison to controls [13], [14]. Little is known about how eIF2B mutations have an effect mainly on white matter (WM). Recent studies suggested a primitive abnormal maturation of glial cells during development in eIF2B-related disorders, leading to the alteration of the WM structure and to associated neurological dysfunctions [15]–[17]. In the present study, we investigated the CSF glycomic profile of control patients at different ages and compared them to those of eIF2B mutated patients using MALDI-TOF/MS (matrix-assisted laser desorption ionisation - time of flight - mass spectrometry) in order to test its usefulness as a biomarker identification in brain developmental disorders. We first described distinct CSF control N-glycan profiles in two groups of patients' age at sampling (before and after 5 years of age). We then identified highly indicative changes in CSF N-glycan profiles of eIF2B-related disorders without changes in plasma. Four of these CSF N-glycan structures appeared as biomarker candidates that characterise the glycomic profiles of eIF2B-related disorders.

## Patients, Materials and Methods

### Patients' samples collection

Studies have been performed with the ethical agreement of the “centre de protection des personnes Sud-Est VI, France” and the Institutional Review Board of the National Institute of Neurological Disorders and Stroke, National Institutes of Health, Bethesda, Maryland, USA. A signed written informed consent was obtained from the patients or their legal guardians. Our ethics committee specifically approved this study. Only excess CSF and plasma drawn for other clinical or research purposes was used for these analyses. Samples from eIF2B-mutated patients came from the French Leukobank, approved by the ethical committee “centre de protection des personnes Sud-Est VI, France”. We obtained written informed consents for this original human work. In total, CSF and EDTA plasma from respectively 12 and 9 eIF2B-mutated patients and from 17 and 15 control individuals without neurologic disorders were collected. The biological fluids were centrifuged at 500×g during 10 min and supernatants were transferred to clean Eppendorf polypropylene tubes and stored at −80°C until analysis.

### Materials

Endoglycosidase, peptide-N-Glycosidase F (PNGase F; EC 3.2.218.3.5.1.52) was from Roche Diagnostics (Meylan, France; www.rochediagnostics.fr), SPE Carbo from GRACE Alltech (Epernon, France; www.grace.com), Sep-Pack Plus C18 were from Waters (St Quentin Yvelines, France; www.waters.com). Methanol, acetonitrile are LCMS grade. Nonidet P40; sodium dodecyl sulphate; 2 mercaptoethanol; DMSO; iodomethane; Sodium hydroxide; TFA, sodium phosphate salts were purchased from SIGMA Aldrich (St Quentin Fallavier, France; www.sigmaaldrich.com). 2, 5-Dihydroxybenzoïc acid and peptide calibrant C104 were purchased from LaserBio Labs (Sophia-Antipolis, France; www.laserbiolabs.com).

### Methods

#### N-glycan release from biological fluids and permethylation

Twenty µl of human plasma and 250 µl of CSF were used. Proteins were denaturated with SDS, deglycosylated using 20 units PNGase F at 37°C and released N-glycans were permethylated according to Morelle et al., 2007 [18].

#### MALDI-TOF MS analyses

Mass spectra of permethylated N-glycans resuspended in 20 µl of 50% methanol/water were acquired in the positive reflector mode (m/z range 1500 to 5000 Da) on MALDI TOF DE PRO (Applied Biosystems, Inc., Framingham,MA) with DHB as matrix (10 mg/ml, ratio 1∶1). External calibration of spectra allowed obtaining a mass accuracy of 20 ppm. MALDI-PSD fragmentation was performed on selected ions to confirm structures.

#### Data evaluation and statistics

MALDI–MS data were processed using DataExplorer 4.0 to generate files listing m/z values and to compare intensities of 34 ions corresponding to the main N-glycan structures from plasma and CSF samples. Interpretation of N-glycan structures corresponding to monoisotopic masses was performed using Expasy GlycoMod tool (http://ca.expasy.org/tools/glycomod/) and Glycoworkbench. Glycans and glycan types (High mannose, complex biantennary, complex triantennary or biantennary with bisecting GlcNAc, fucosylated, sialylated and hybrid) were compared between groups using ANOVA. Selected ions were fragmented by MALDI-PSD to further elucidate the corresponding glycan structures. Since a clear distinction of relative intensities of the isobaric complex bisecting and triantennary structures was not possible, these structures were not differentiated in the quantitative test. A difference between two groups of data was considered statistically significant when p-values were less than 0.005. Receiver Operating Characteristics (ROC) curves were used to assess the sensitivity and specificity of potential diagnostic variables with ANOVA p-values<0.001. Analyses were performed using SAS ver. 9.1.3. A principal component analysis was also performed according to TANAGRA [19].

## Results

### Establishment of N-glycan profiles in human control CSF

We established MALDI-TOF MS spectra of permethylated N-glycans from 17 control CSF, age at sampling from 1.5 to 50 years (Table 1), with the aim to elucidate the N-glycosylation profile and to generate reference spectra from CSF of healthy donors.

**Table 1:** Patients' samples used in this study: Clinical and biological information were added for each patient.

Patient number	Sex	Status	Age at disease onset (y)	Age at sampling (y)	Biological fluid	Mutated gene	Mutation protein	GEF activity (% of controls)
VIC-WAL	F	C	-	3	P	-	-	NA
EMM-GRA	F	C	-	6	P	-	-	NA
G1498	F	C	-	9	P	-	-	NA
CR10	M	C	-	10	P	-	-	NA
CR18	M	C	-	18	P	-	-	NA
MAR-TAN	F	C	-	18	P	-	-	NA
GUI-BRI	M	C	-	20	P	-	-	NA
ANN-FOG	F	C	-	28	P	-	-	NA
NIC-LUG	M	C	-	28	P	-	-	NA
G1904-3	F	C	-	30	P	-	-	NA
FRE-BOT	M	C	-	32	P	-	-	NA
LAU-CHE	M	C	-	33	P	-	-	NA
NAT-NER	F	C	-	39	P	-	-	NA
CHR-JAN	M	C	-	53	P	-	-	NA
G2081-4	M	C	-	58	P	-	-	NA
G928-1	F	A	3	8	P	*EIF2B5*	p.Arg113His/p.Arg113His	49±3
G73-1	M	A	4	16	P+Profile II CSF	*EIF2B5*	p.Arg113His/p.Arg113His	70.8±7
G630-1	M	A	4.5	20 & 15	P+Profile II CSF	*EIF2B5*	p.Arg113His/p.Arg113His	77.5±2.5
*G648-2	M	A	7	20	P+Profile II CSF	*EIF2B2*	p.Glu213Gly/p.Glu213Gly	64±4
G648-1	M	A	7	23	P+Profile II CSF	*EIF2B2*	p.Glu213Gly/p.Glu213Gly	59±1
G736-1	F	A	8	11	P+Profile II CSF	*EIF2B4*	p.Arg374Cys/p.Arg374Cys	80
G944-1	F	A	10	38	P+Profile II CSF	*EIF2B2*	p.Ser171Phe/p.Met203fs	NA
G1014-1	F	A	16	16	P	*EIF2B5*	p.Arg113His/p.Arg195Cys	68±4
G904-1	F	A	2	8	P	*EIF2B5*	p.R113H/ p.G481fs493stop	70±1.2
G1693-1	F	C	-	1.5	Profile I CSF	-	-	NA
G1282-1	F	C	-	2	Profile II CSF	-	-	NA
G1301-1	F	C	-	2	Profile I CSF	-	-	NA
G1328-1	M	C	-	3	Profile I CSF	-	-	NA
G1728-1	M	C	-	4	Profile I CSF	-	-	NA
G1208	M	C	-	5	Profile I CSF	-	-	NA
YOU-LAM	M	C	-	5	Profile II CSF	-	-	NA
CR3.5	F	C	-	3.5	Profile II CSF	-	-	NA
G791-1	F	C	-	7	Profile II CSF	-	-	NA
VIR-DEL	F	C	-	8	Profile II CSF	-	-	NA
CR8	F	C	-	8	Profile II CSF	-	-	NA
CR9	F	C	-	9	Profile II CSF	-	-	NA
CR11	F	C	-	11	Profile II CSF	-	-	NA
MOR-FOL	F	C	-	12	Profile II CSF	-	-	NA
CR15	M	C	-	15	Profile II CSF	-	-	NA
CR39	F	C	-	39	Profile II CSF	-	-	NA
BER-NAR	M	C	-	50	Profile II CSF	-	-	NA
G590-2	F	A	1	3.5	Profile II CSF	*EIF2B5*	p.Tyr343Cys/p.Ile385Val	45.8±2.2
G590-1	F	A	3	9	Profile II CSF	*EIF2B5*	p.Tyr343Cys/p.Ile385Val	44.9±4.3
G954-1	M	A	3.5	8	Profile II CSF	*EIF2B5*	p.Arg113His/p.Arg269Leu	NA
G522-1	F	A	3.5	11	Profile II CSF	*EIF2B5*	p.Ala16Asp/p.Arg113His	54±6
G1008-1	F	A	4	25	Profile II CSF	*EIF2B5*	p.Arg113His/p.Arg113His	76.1±2.6
G984-1	F	A	5	12	Profile II CSF	*EIF2B2*	p.Glu213Gly/p.Glu213Gly	51.5±0.5

M: male; F: female.

C: control; A: affected with eIF2B-related disorders.

NA: not available.

P: EDTA-plasma; CSF: cerebrospinal fluid;

* Patient previously described in Fogli et al 2004 [13] and Vanderver et al. 2008 [5].

**Figure 1:**
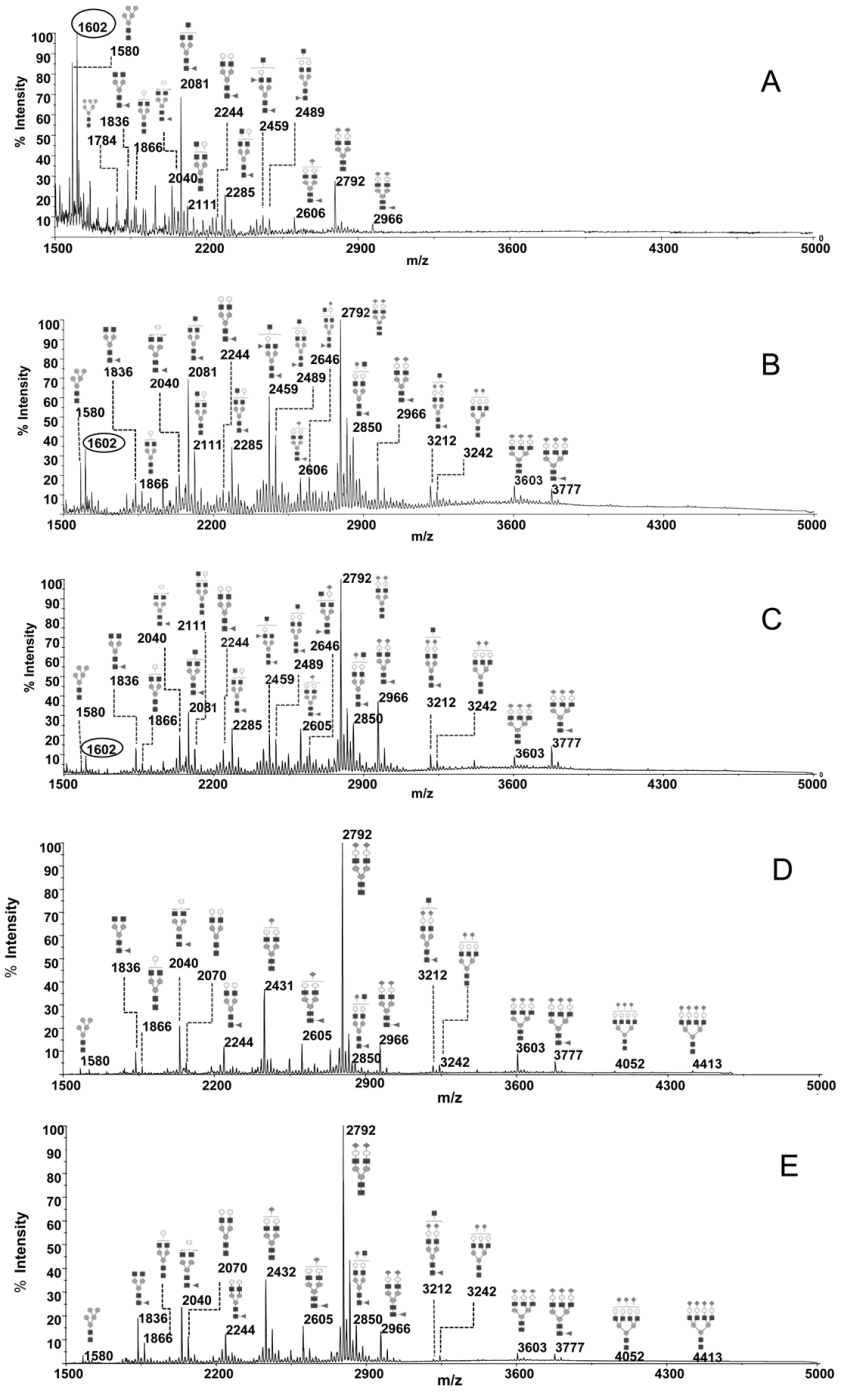
MALDI spectra of permethylated N-glycans derived from plasma and CSF from control or affected eIF2B-mutated patients. **A)** Profile I CSF from control patient (G1693); **B**) Profile II CSF from control patient (CR15); **C**) CSF from eIF2B-mutated patient (G630-1). **D**) Plasma from control patient (NIC LUG 28 years); **E**) Plasma from the eIF2B-mutated patient (G630-1). The other structures not annoted here due to the complexity of the Figure are listed in Table S1. Fucose (Fuc): triangle, Mannose (Man): grey circle, N-acetyl glucosamine (GlcNAc): square, Galactose (Gal): white circle; Sialic acid (NeuAc): lozenge.

250 µl of each CSF were sufficient to generate quantifiable permethylated N-glycan profiles, over an m/z range of 1500–5000, with a good signal to noise ratio. Two types of CSF control MALDI spectra were obtained showing distinct intensities of ions. The first type, named “profile I”, showed major ions at m/z 1602, 1580, 2081, and was found for 5 individuals with age at sampling between 18 months and 5 years of age (Figure 1A, Table 1, Table S1 for individual values). In contrast, the second type, named “profile II”, contained dominant ions at m/z 2081, 2459, 2489, 2792, 2850 and was obtained for 12 individuals with age at sampling between 2 and 50 years (Figure 1B, Table 1, Table S1 for individual values).

**Figure 2:**
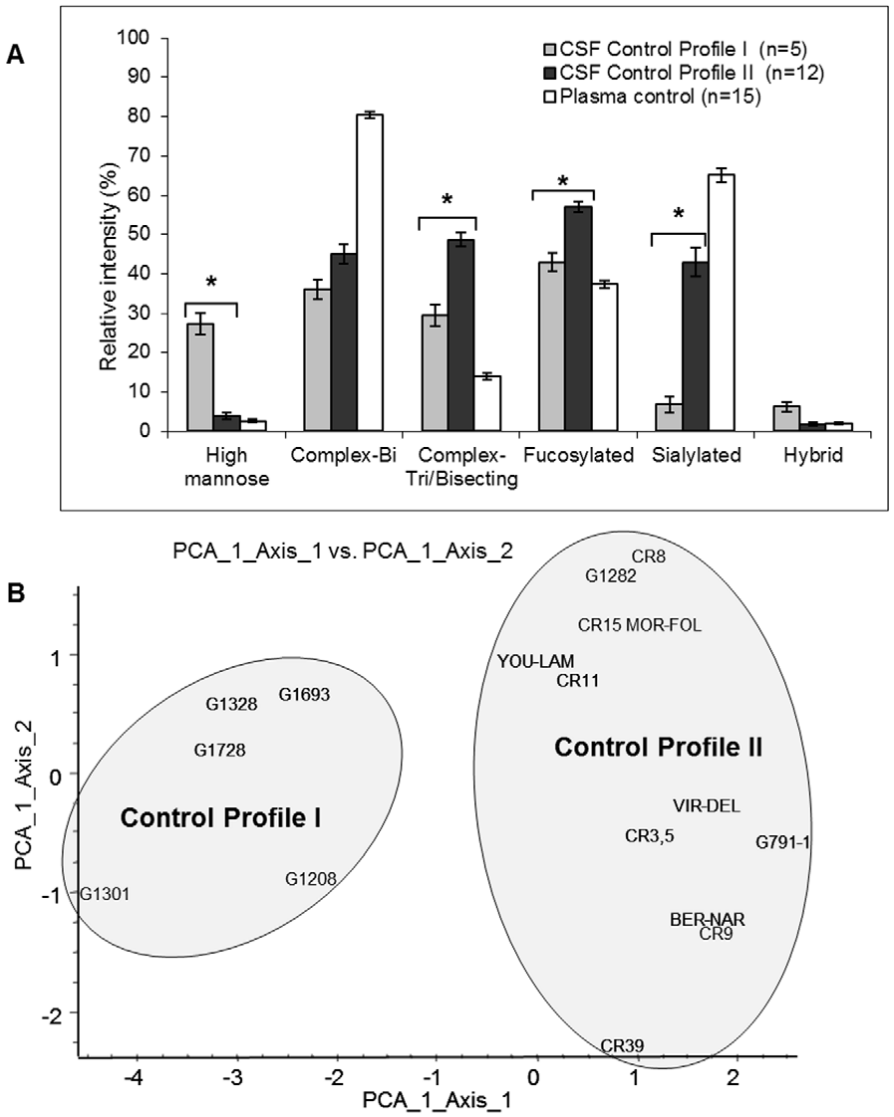
Two distinct N-glycan profiles exist in control CSF and are different to N-glycan profiles from plasma. **A**) Comparison in the relative intensities (%, +/−SEM) of N-glycan types from control plasma and control CSF. **B**) Principal component analysis illustrating the differences between control CSF profiles leading to the distinction of two profiles named profile I and profile II. * Statistically significant with ANOVA test *p* values<0.005.

In order to further characterize these differences, relative intensities of a total of 34 individual N-glycans falling into 6 separate N-glycan families were determined for each MALDI spectra. The relative intensities of glycans within these N-glycan families were calculated for both profiles and statistically significant differences could be detected (Figure 2A). Principal component analysis showed two distinct N-glycan profiles among the population of 17 control CSF (Figure 2B). A 7-fold decrease between profile I (Figure 1A) and II (Figure 1B) in high mannose type glycans (27.3±2.7% *versus* 3.9±0.8%) appeared statistically significant (p = 0.0016). The most abundant structure in profile I CSF was high mannose type structure (Man)_5_ (GlcNAc)_2_ (at m/z 1580). Hybrid glycan structures decreased threefold. Profile II CSF was characterised by an increase in complex tri-antennary/bisecting (29.6±2.7% versus 48.6±1.7%) (p = 0.0016) and fucosylated type structures (43.0±2.3% *versus* 57.0±1.4%) (p = 0.0022). A 6-fold increase in sialylated *N*-glycan structures (6.8±2.1% *versus* 43.0±3.7%) (p = 0.0016) also distinguishes the two profiles. In contrast, complex bi-antennary type structures showed no significant difference between the two profiles.

MALDI-TOF spectra revealed the presence of an intense ion at m/z 1602 for profile I control CSF (Figure 1A), which is also present with lesser intensity in profile II CSF (Figure 1B) but a classical (Man)_3_ (GlcNAc)_2_ core containing *N*-glycan could not be assigned to this ion. MALDI-PSD fragmentation (Figure S1) allowed to identify the structure as a linear glycan fragment (NeuAc)_3_ (Gal)_1_ (GlcNAc)_1_, corresponding probably to a degradation fragment of polysialic acid (PSA)-containing *N*-glycans, found on glycoproteins, such as NCAM, within the CNS.

After having established CSF reference profiles, glycoprofiling of samples from eIF2B-mutated patients was performed and spectra were compared to their respective reference.

### Identification of quantitative variations of specific N-glycans within overall N-glycan profiles from CSF of eIF2B-mutated patients

We established MALDI-TOF MS profiles of permethylated N-glycans from 12 CSF samples of patients with eIF2B-related disorders (age at sampling from 3.5 to 39 years) (Table 1).

**Figure 3:**
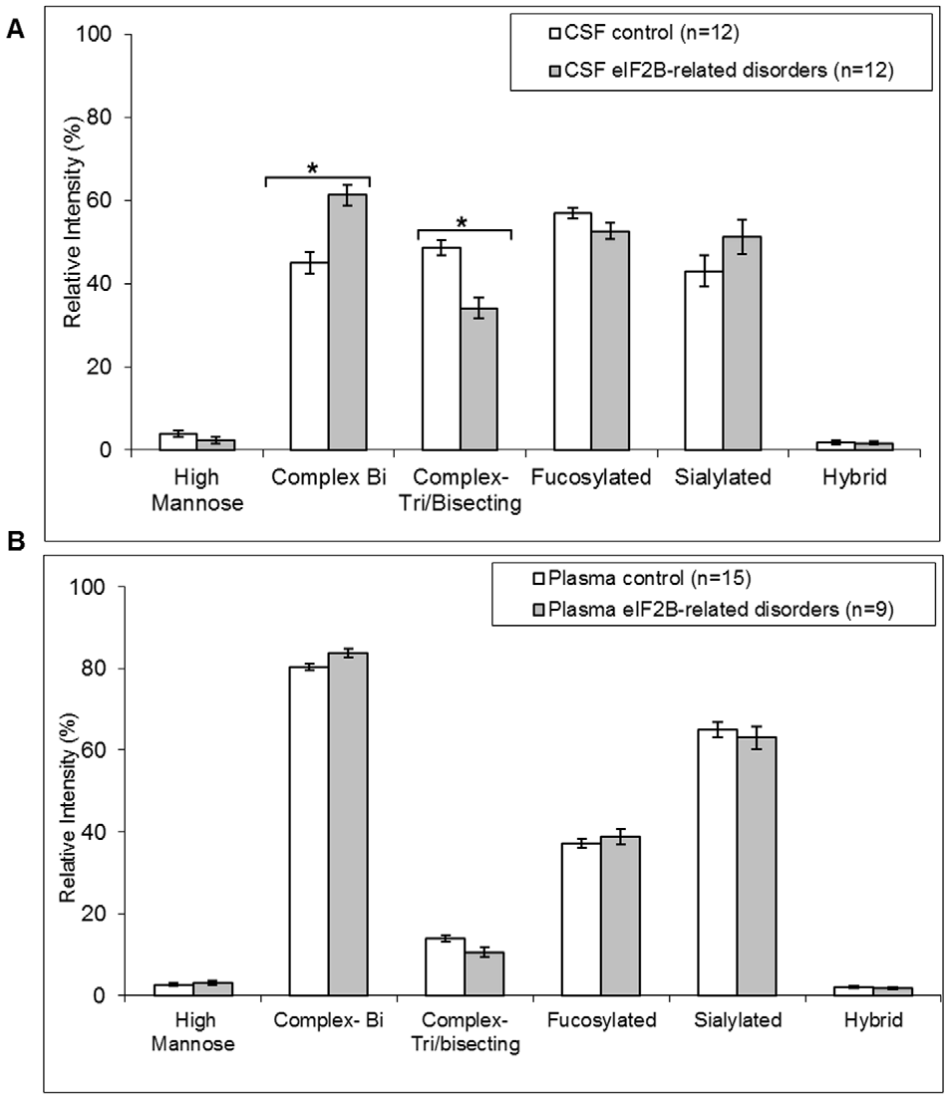
Comparison of relative intensities (%, +/−SEM) of N-glycan types derived from CSF (A) or plasma (B) from healthy individuals (controls) and affected eIF2B-mutated patients. * Statistically significant with ANOVA test *p* values<0.0001.

The 12 spectra were characterised by the dominance of the complex biantennary disialylated glycan at m/z 2792 and very low levels of the ion at m/z 1580 (Figure 1C, Table 1, Table S1 for individual values). Patient N-glycan profiles were therefore compared to control profile II CSF of 12 age and sex matched controls. An increase in the relative intensity of complex bi-antennary structures (61.3±2.4% *versus* 45.0±2.5%) (p<0.0001) and the concomitant decrease of complex tri-antennary/bisecting structures (34.2±2.4% *versus* 48.6±1.7%) (p<0.0001) which are generally considered as brain or CNS type glycan structures were consistent among all samples (Figure 3A). PSD fragmentation of the ion at m/z 2081 (GlcNAc)_3_ (Fuc)_1_ (Man)_3_ GlcNAc)_2_ reveals a fragment ion at Y1372 specific for a tri-antennary glycan (Figure S1). PSD fragmentation of ion at m/z 2459 (Gal)_1_ (GlcNAc)_3_ (Fuc)_2_ (Man)_3_ GlcNAc)_2_ reveals the presence of fragment ions at m/z B486 and Y1996 specific for a presence of bisecting structures but also a minor fragment ion at Y1751 suggesting the presence of tri-antennary glycans (Figure S1). Thus these results suggest a major but not exclusive presence of bisecting N-glycans corresponding to peaks at m/z 2081 and 2459 within the CSF glycome. Fragmentation of the ion at m/z 2459 clearly indicates the presence of product ions B 660 and Y 1822 that are characteristic for the Lewis type antennary structure (Gal)_1_ (GlcNAc)_1_ (Fuc)_1_- (Figure S1). No variations in fucosylation, sialylation, hybrid and high mannose types were detected.

**Figure 4:**
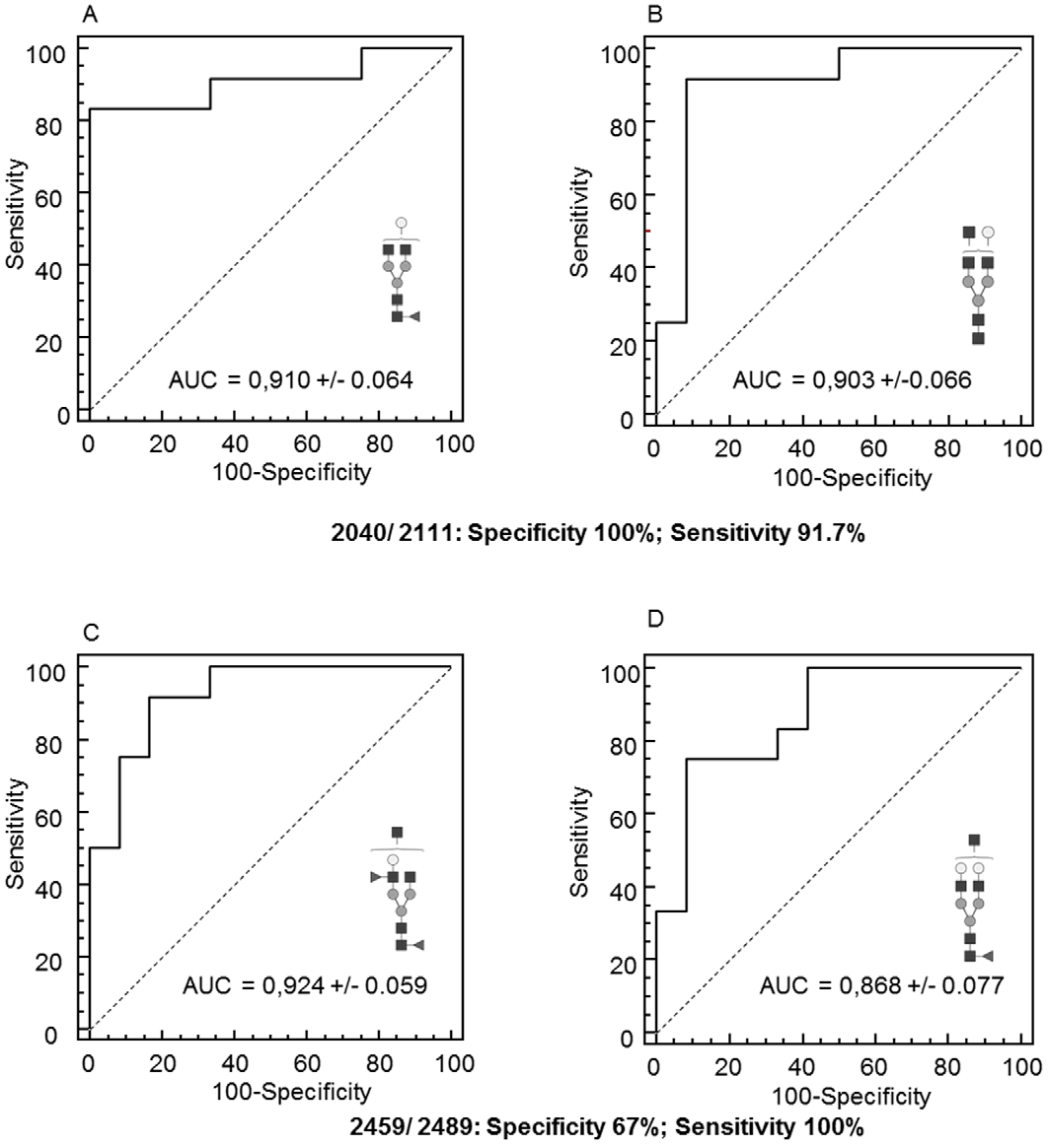
Representative ROC curve analysis AUC plots performed for different CSF N-glycan structures (ANOVA *p* values<0.001): m/z 2040 (A), 2111 (B), 2459 (C) and 2489 (D).

Relative intensity of individual N-glycans showed statistically relevant differences for 11 structures (p values<0.05) between patient and control CSF (Table 2). Three glycan structures (at m/z 2111, 2459 and 2489) corresponding to complex tri-antennary/bisecting N-glycans decreased significantly within CSF from eIF2B-mutated patients (p values<0.001). The fucosylated bi-antennary structure at m/z 2040 increased significantly with the pathology (p value<0.001) (Table 2). ROC test AUC values were calculated for these four N-glycan structures (Figure 4A–D). Glycan structure at m/z 2040 is more specific (specificity = 100%, sensitivity = 83.3 ; cut-off value >5.39), glycan structure at m/z 2111 gave 91.7% sensitivity and specificity for cut-off value ≤2.94 and association of N-glycans at m/z 2040 and m/z 2111 gave 100% specificity and 91.7% sensitivity; in the same way, 100% sensitivity was obtained by association of glycans at m/z 2459 (Se = 91.7, sp = 83.3, cut-off value ≤4.75) and m/z 2489 (Se = 75.0, sp = 91.7, cut-off value ≤3.24) with 67% specificity making these structures predictive of the disease state.

**Table 2:** Relative intensities of 11 *N*-glycans from CSF samples.

		eIF2B-related disorders (n = 12)	Controls (n = 12)	*ANOVA*
N-glycan	m/z	Relative intensity (%)+/−SEM	Relative intensity (%)+/−SEM	*p* values
(GlcNAc)_2_+(Man)_3_(GlcNAc)_2_	1662	0.8+/−0.2	1.9+/−0.4	0.0229
(GlcNAc)_2_ (Fuc)_1_+(Man)_3_(GlcNAc)_2_	1836	5.0+/−0.5	3.5+/−0.5	0.0474
(Gal)_1_ (GlcNAc)_2_ (Fuc)_1_+(Man)_3_(GlcNAc)_2_	2040	7.9+/−1.0	4.1+/−0.3	0.0009
(Gal)_2_ (GlcNAc)_2_ (Fuc)_1_+(Man)_3_(GlcNAc)_2_	2244	3.7+/−0.5	2.2+/−0.2	0.0122
(NeuAc)_2_ (Gal)_2_ (GlcNAc)_2_+(Man)_3_(GlcNAc)_2_	2792	25.9+/−3.1	16.0+/−1.9	0.0136
**(GlcNAc)_3_ (Fuc)_1_+(Man)_3_(GlcNAc)_2_**	**2081**	7.4+/−0.8	10.9+/−0.8	0.0050
**(Gal)_1_ (GlcNAc)_3_+(Man)_3_(GlcNAc)_2_**	**2111**	2.3+/−0.3	4.5+/−0.3	<0.0001
**(Gal)_2_ (GlcNAc)_3_+(Man)_3_(GlcNAc)_2_**	**2315**	0.9+/−0.2	1.9+/−0.3	0.0128
**(Gal)_1_ (GlcNAc)_3_ (Fuc)_2_+(Man)_3_(GlcNAc)_2_**	**2459**	3.3+/−0.4	6.9+/−0.6	<0.0001
**(Gal)_2_ (GlcNAc)_3_ (Fuc)_1_+(Man)_3_(GlcNAc)_2_**	**2489**	3.1+/−0.2	4.6+/−0.3	0.0007
**(NeuAc)_1_ (Gal)_1_ (GlcNAc)_3_ (Fuc)_1_+(Man)_3_(GlcNAc)_2_**	**2646**	2.0+/−0.3	3.0+/−0.3	0.0182

Relative intensities of the 11/34 *N*-glycans from CSF samples with ANOVA test *p* values<0.05, five complex bi-antennary structure, and six complex tri-antennary/bisecting structures (in bold).

Measuring relative intensity variation of these four N-glycan structures in CSF glycan profiles as potential biomarkers for identifying eIF2B-related disorders is strongly suggested. As lumbar puncture is more invasive than a simple blood sampling, we decided to investigate whether the observed differences in CSF N-glycan profiles were also found in eIF2B-mutated patients' plasma.

### Absence of quantitative variations of specific N-glycan profiles in plasma of eIF2B-mutated patients

MALDI-TOF MS profiles of permethylated N-glycans from 8 plasma samples from patients with eIF2B-related disorders (age at sampling from 8 to 38 years) were compared to 15 healthy age and sex matched controls (age at sampling from 3 to 58 years) (Table 1).

Twenty µl of plasma were sufficient to generate permethylated N-glycan profile MS spectra with a good signal to noise ratio over an m/z range of 1500–5000. N-glycan profiling of plasma from control and eIF2B mutated individuals resulted in highly reproducible spectra showing the specific profiles represented in Figure 1D and E. Comparison of plasma spectra from controls and eIF2B-mutated patients showed no differences (compare Figure 1D and 1E). The comparison of relative intensities of each N-glycan structure and of glycan families confirms the absence of significant differences between controls and affected samples (Figure 3B).

We thus show that the differences in N-glycan profiles found in CSF from eIF2B-mutated patients as compared to control CSF are specific for this biological fluid and cannot be detected in corresponding plasma samples. We also did not find any other differences when comparing N-glycan profiles from mutated and control plasma.

## Discussion

Using MALDI TOF MS-based analysis of permethylated glycans, we discovered an evolution of the control CSF glycome from N-glycan profiles rich in high mannose type structures to profiles with complex sialylated structures that could be correlated with postnatal brain maturation. We identified significant disease correlated differences in CSF but not in plasma N-glycan profiles of eIF2B-mutated patients suggesting for the first time a global change in N-glycosylation of CSF proteins in this pathology.

The MALDI-TOF-based glycoprofiling method has been used previously to identify and quantify N-glycan structures in glycomic studies [20] and to detect rapidly specific N-glycan biomarkers in plasma or serum of patients affected by different forms of cancer or type II CDG [21]–[28].

The analysis of CSF control samples led to the discovery of a dynamic progression of CSF N-glycan profiles. Profiles I were found in control CSF from young patients, with age at sampling between 1.5 and 5 years, and may correspond in the brain to the achievement of the intense primitive myelinating phase associated with active synaptogenesis. Profiles II were found in control CSF with age at sampling between 2 and 50 years. An overlap between the two CSF glycan profiles was observed between 2 and 5 years, suggesting that inter-individual variations might occur during brain development and might lead to different delays in N-glycosylation dynamics. Increased sampling with CSF collected between 2 and 6 years and before 1 year of age would be useful to delineate this interphase more precisely. For this study it was not necessary to pool CSF samples from several donors as it was previously described for HPLC based glycoprofiling of CSF [29]. Previous studies already described age-dependent galactosylation in serum IgG, or N-glycomics changes in serum proteins during human aging [30]–[32]. This is the first time that a dynamic and developmental profile-dependent evolution of N-glycosylation in normal CSF has been described. The significance of its correlation with brain maturation and development has to be further elucidated. It is known that glycosylation of specific CNS proteins plays a crucial role in establishing and maintaining the tight interactions that exist between glial cells and neurons, thus enabling cerebral plasticity. In this context, it is interesting to note the presence of the glycan fragment (NeuAc)_3_ (Gal)_1_ (GlcNAc)_1_ in CSF, which might correspond to a degradation product of polysialic acid (PSA)-containing N-glycans. This type of glycans is notably found on glycoproteins, such as NCAM, that are highly implicated in the development and the formation of plasticity of the CNS [33].

The permethylated *N*-glycan profiles obtained from 12 eIF2B-mutated CSF samples corresponded exclusively to profile II and were therefore compared to profile II references. Patient samples showed a significant decrease of tri-antennary/bisecting structures and an increase in bi-antennary glycans. Within these global CSF N-glycan profiles the glycan structure at m/z 2081 decreases while the sialylated structure at m/z 2792 increases. Knowing that these structures are the main glycans found on transferrin from CSF, we can conclude that their quantitative variations between eIF2B-mutated and control CSF samples are in accordance with the findings of Vanderver and coll (2005, 2008) concerning a decrease of the asialotransferrin/total transferrin ratio in eIF2B-mutated CSF [5], [6]. Our results suggest that the differences in glycosylation described by these authors for CSF derived transferrin are representative of more global changes in N-glycosylation of proteins that are related to the pathology. In this respect it might be interesting to analyse N-glycosylation profiles of other, less abundant glycoproteins within the CSF.

A complex monogalactosyl biantennary fucosylated glycan (at m/z 2040) increased with the pathology and appeared as potentially promising marker candidate. Knowing that this structure is a dominant N-glycan on IgG which are quantitatively important in biological fluids, we could suspect an increase in IgG in CSF of patients. Clinical presentation and CSF protein analyses do not support a supposed inflammation. A contamination of eIF2B-mutated CSF by plasma during lumbar puncture was also excluded as the ion at m/z 2431, second major N-glycan present in plasma, was stable (3.3±0.2%) in N-glycan profiles of patient and control CSF. The three other potentially promising N-glycan marker candidates are GlcNAc3 containing triantennary/bisecting type structures at m/z 2111, 2459 and 2489 (Table 2). One eIF2B-mutated patient (G984-1), with a classical phenotype, was not detected by this technique. This patient exacerbated a significant decrease of eIF2B GEF activity (<77.5%) (Table 1) [13].

The observed quantitative variations within CSF glycan profiles might be correlated to a down-regulation on the translational level of the expression of specific enzymes involved in glycan biosynthesis influenced by the mutation of translation initiation factor eIF2B. A decrease in the activity of beta-1.4-mannosyl-glycoprotein 4-beta-N-acetylglucosaminyltransferase III (GlcNAc-T III) which is responsible for the synthesis of bisecting glycans, might thus explain the observed reduced levels of triantennary/bisecting structures. Stanta et coll. (2010) described a comparable correlation between down-regulation of the MGAT3 gene encoding GlcNAc-T III and other genes on the transcriptional level and variations of the CSF glycome related to Schizophrenia. [29] Further analyses of the potential influences of eIF2B mutation in eIF2B-related disorders on the expression of enzymes within the N-glycan biosynthesis pathway will be necessary to elucidate their eventual correlation to the observed changes within the CSF N-glycan profiles.

Based on our results, an association of this glycomic approach and the determination of GEF activity in patients' lymphoblasts can be used to screen patients with 100% specificity and 100% sensitivity before sequencing or not the five EIF2B genes. In a patient diagnostic setting, we propose to first measure the eIF2B GEF activity in lymphoblastoid cell lines from patients selected based on MRI and clinical symptoms to carry potentially eIF2B-related disorder. If the GEF activity is >77.5% in patients with a classic or moderate clinical form of eIF2B-related disorders, analyses of CSF glycome will be useful to validate or not the eIF2B-related disorders. In order to enhance clinical value of such glycan analyses, perspective of this work will be to analyse CSF glycan profiles from patients with other white matter disorders not linked to eIF2B mutations.

Analysis of the recent *Eif2b5* knock in (KI) mice model of the pathology reported a delay in brain WM development [16], and functional studies performed in glial cell *in vitro* models or in patients' brains showed a defective glial maturation with a significant increase in premyelinating oligodendrocyte progenitor proliferation and a defect in the maturation of astrocytes [15]–[17]. Knowing the key role of N-glycosylation in WM cohesion and function, we hypothesize that the N-glycosylation abnormalities we observed in patients' CSF may participate in the glial cell maturation defects reported in eIF2B-related disorders. Finally, the present report suggests a potential interest of CSF N-glycan profile analysis for other neurological disorders involving gliogenesis/synaptogenesis changes.

## Supporting Information

Table S1 **Relative intensities for the 34 m/z corresponding to N-glycans for 24 individuals, the average for 12 control samples and 12 eIF2B-mutated samples with the ANOVA p values.** In grey, the 4 structures with p values<0.001.
(DOCX)

Figure S1 **MALDI-PSD fragmentation of selected glycan molecules.** MS peaks corresponding to specific fragment ions are indicated within each of the three spectra and characteristic fragmentation patterns are annotated for each of the glycan structures. For figures B and C both bisecting and triantennary structures are annotated with fragment ions in the spectra potentially indicating the presence of both types of glycan structures within the corresponding mass peak. A) PSD fragmentation of the ion at m/z 1602 (NeuAc)_3_ (Gal)_1_ (GlcNAc)_1_ indicates the presence of a linear glycan fragment probably originating from PSA glycans. B) Fragmentation of the ion at m/z 2081 (GlcNAc)_3_ (Fuc)_1_ (Man)_3_ (GlcNAc)_2_ and annotation of the corresponding triantennary and bisecting glycan structures. C) Fragmentation of the ion at m/z 2459 (Gal)_1_ (GlcNAc)_3_ (Fuc)_1_ (Man)_3_ (GlcNAc)_2_ and annotation of the corresponding triantennary and bisecting structures showing the presence of the Lewis type antennary structure (Gal)_1_ (GlcNAc)_1_ (Fuc)_1_ represented by specific fragmentation ions B 660 and Y 1822.
(DOCX)
