# Supplementary figures and images for: CSF N-Glycan Profiles to Investigate Biomarkers in Brain Developmental Disorders: Application to Leukodystrophies Related to eIF2B Mutations
Source: PLoS One. 2012 Aug 29;7(8):e42688. doi: 10.1371/journal.pone.0042688 (PMC3430715; doi:10.1371/journal.pone.0042688)

Figure S1


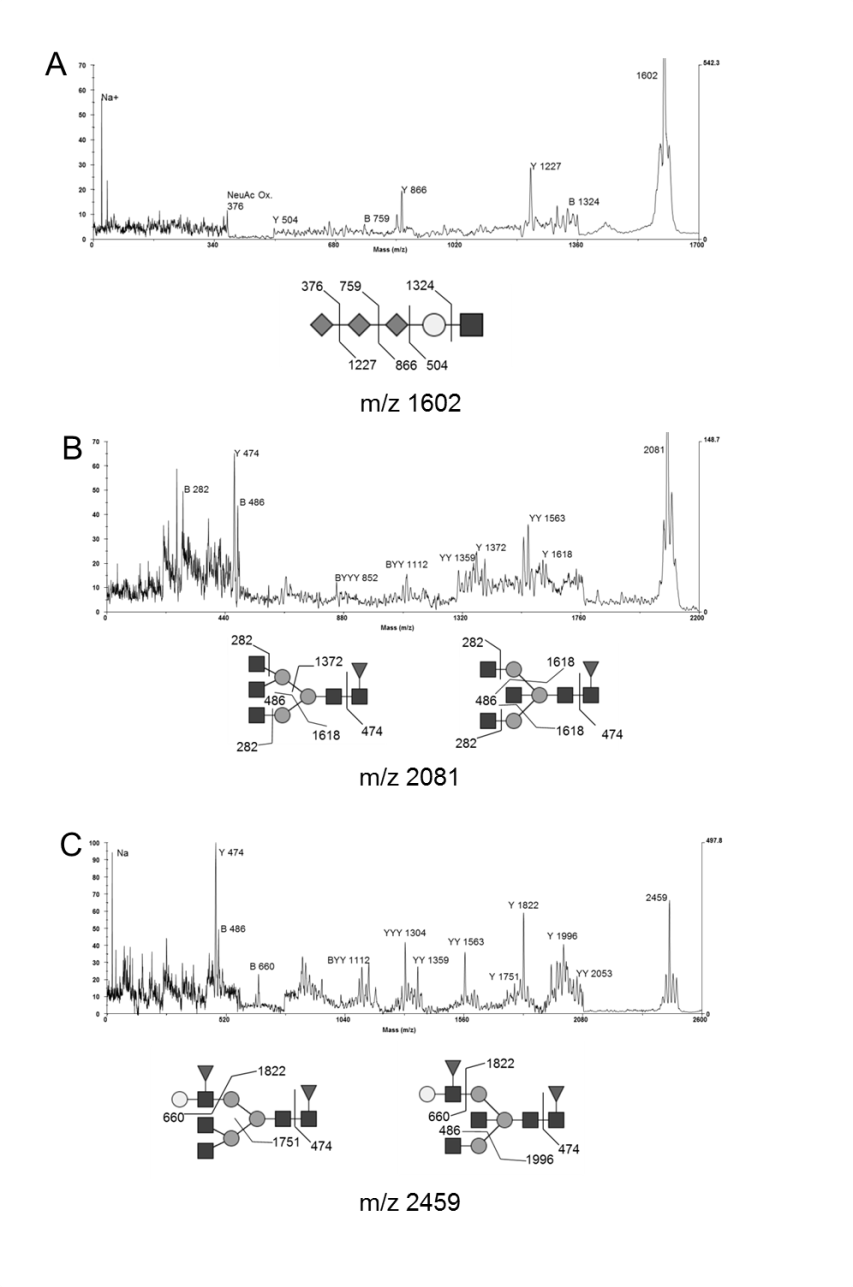

Supplement: Figure S1 — MALDI-PSD fragmentation of selected glycan molecules. MS peaks corresponding to specific fragment ions are indicated within each of the three spectra and characteristic fragmentation patterns are annotated for each of the glycan structures. For figures B and C both bisecting and triantennary structures are annotated with fragment ions in the spectra potentially indicating the presence of both types of glycan structures within the corresponding mass peak. A) PSD fragmentation of the ion at m/z 1602 (NeuAc)3 (Gal)1 (GlcNAc)1 indicates the presence of a linear glycan fragment probably originating from PSA glycans. B) Fragmentation of the ion at m/z 2081 (GlcNAc)3 (Fuc)1 (Man)3 (GlcNAc)2 and annotation of the corresponding triantennary and bisecting glycan structures. C) Fragmentation of the ion at m/z 2459 (Gal)1 (GlcNAc)3 (Fuc)1 (Man)3 (GlcNAc)2 and annotation of the corresponding triantennary and bisecting structures showing the presence of the Lewis type antennary structure (Gal)1 (GlcNAc)1 (Fuc)1 represented by specific fragmentation ions B 660 and Y 1822. (DOCX) [file pone.0042688.s002.docx]
